# Prognostic significance of isolated tumor cells and nodal tumor burden in endometrial cancer: a population-based cohort study

**DOI:** 10.2340/1651-226X.2026.45813

**Published:** 2026-08-11

**Authors:** Andrei Chilianu, Charlotta Riese, Mahmood Ul Hassan, Jaiteh Darbo, Henrik Falconer, Linda Eriksson, Sahar Salehi

**Affiliations:** a Department of Women’s and Children’s Health, Karolinska Institutet, Stockholm, Sweden; b Department of Pelvic Cancer, Theme Cancer, Karolinska University Hospital, Stockholm, Sweden; c Department of Oncology-Pathology, Karolinska Institutet, Stockholm, Sweden; d Division of Biostatistics, Institute of Environmental Medicine, Karolinska Institutet, Stockholm, Sweden

**Keywords:** endometrial neoplasms, sentinel lymph node biopsy, lymphatic metastases, micrometastases, isolated tumor cells, prognosis, survival analysis

## Abstract

**Background and purpose:**

Since the introduction of sentinel lymph node biopsy, nodal assessment has expanded to nearly all women with endometrial cancer. However, evidence on the prognostic significance of isolated tumor cells (ITCs) and nodal tumor burden remains limited. We therefore investigated the association of ITCs and nodal tumor burden with survival.

**Patients and methods:**

This cohort study included women with endometrial cancer who underwent surgery in Region Stockholm–Gotland, Sweden, between 2010 and 2024, and either underwent sentinel lymph node biopsy or were registered as having lymph node metastases (FIGO stage IIIC). Nodal dissemination was categorized as bulky, macro-, micro-metastases, or ITCs. Overall survival (OS) and cancer-specific survival (CSS) were evaluated using multivariable Cox regression in two separate analyses: (1) ITCs versus node-negative patients and (2) nodal tumor burden (bulky, macro-, micro-metastases) among node-positive patients.

**Results:**

Among 1219 patients, 310 (25%) had nodal dissemination, including 250 with nodal metastases and 60 with ITCs. No significant difference in OS was observed between patients with ITCs and node-negative patients (Hazard ratio [HR] 1.94, 95% confidence interval [CI] 0.75–5.06). Adjuvant therapy was more frequent among patients with ITCs (47% vs. 11%).

In patients with nodal metastases, no clear OS differences were observed by nodal burden. Compared with micrometastases, survival was similar for macrometastases (HR 0.95, 95% CI 0.50–1.81) and bulky metastases (HR 1.19, 95% CI 0.64–2.21). Age > 68 years and p53 mutation were associated with worse survival. CSS analyses yielded similar findings, with no significant differences observed in either comparison.

**Interpretation:**

Neither ITCs nor greater nodal burden was clearly associated with survival after adjustment for clinical and molecular factors. Survival appeared to be influenced by patient and tumor characteristics, although estimates were imprecise and modest associations cannot be excluded.

## Introduction

Endometrial cancer is the most common gynecologic malignancy in developed countries and is generally associated with an excellent prognosis [1–3]. Nevertheless, survival varies according to several clinicopathologic factors, including the presence and extent of extrauterine dissemination, particularly lymph node involvement [4, 5]. While nodal positivity confers a worse prognosis, the clinical significance of nodal tumor burden ranging from bulky clinically apparent metastases to macro- and micro-metastases remains unclear. This contrasts with other solid tumors, in which the extent of nodal metastatic dissemination directly informs staging [6–8].

Despite two randomized phase III trials demonstrating no survival benefit of systematic lymphadenectomy in patients considered at increased risk – using heterogeneous definitions – lymph node assessment has become common clinical practice [9, 10]. Moreover, sentinel lymph node biopsy has emerged as the preferred method for nodal assessment and is now widely implemented to guide adjuvant treatment decisions [11–13]. The introduction of sentinel lymph node biopsy has fundamentally altered surgical staging in endometrial cancer, as lymph node assessment – despite lacking evidence of a therapeutic survival benefit – has expanded from selected high-risk patients to nearly all women with endometrial cancer [3]. Moreover, ultrastaging has led to the identification of a new category of lymph node dissemination, isolated tumor cells (ITCs) [14].

The clinical relevance of ITCs remains uncertain. Their detection has resulted in increased use of adjuvant therapy associated with short- and long-term toxicity, despite limited evidence that such treatment improves survival [15–19]. Recent large cohort studies and meta-analyses suggest no survival difference between patients with ITCs and those without lymph node metastases; however, the available evidence remains heterogeneous, and population-based evidence remains limited, particularly from Nordic cohorts [20, 21].

Taken together, management of endometrial cancer – despite its high prevalence – still relies on limited evidence regarding the clinical value of detecting very low-volume nodal disease introduced by contemporary staging practices. The prognostic significance of ITCs and nodal tumor burden remains uncertain, and population-based studies evaluating both within the same cohort remain scarce.

For this reason, the objective of this study was to evaluate the prognostic significance of detecting ITCs in patients with endometrial cancer undergoing sentinel lymph node biopsy. Moreover, in a separate analysis, we investigated the prognostic significance of nodal tumor burden among patients with nodal metastases in a large population-based Swedish cohort.

## Patients and methods

This observational cohort study included women diagnosed with endometrial cancer in the Stockholm–Gotland region of Sweden between January 1, 2010 and June 19, 2024, who fulfilled one of two predefined study inclusion criteria and who underwent primary surgery with curative intent. The region comprises approximately 2.4 million inhabitants, and since 2004, all gynecologic cancer care has been centralized at Karolinska University Hospital, a tertiary referral center. Cancer care in Sweden is publicly funded and universally accessible.

The study followed the Strengthening the Reporting of Observational Studies in Epidemiology (STROBE) guidelines [22].

### Data sources and extraction

Data were obtained from the Swedish Quality Registry of Gynecologic Cancer (SQRGC), a national prospective clinical registry established in 2008 that includes detailed information on diagnosis, staging, treatment, and outcomes. The study cohort was specifically assembled according to two predefined inclusion criteria and therefore did not represent all patients who underwent surgical lymph node staging during the study period. Eligible patients were subsequently identified from the SQRGC according to these criteria. Registry coverage is high and has been validated through linkage with the National Cancer Register. The endometrial cancer module was established in 2010. Vital status and date of death were obtained through linkage with the Swedish Population Registry. All variables were validated against hospital medical records, and predefined variables not captured in the SQRGC were extracted using standardized procedures.

### Inclusion and exclusion criteria

Patients were eligible if they were aged ≥ 18 years, had histologically confirmed endometrial cancer diagnosed between 2010 and 2024, and underwent primary surgery with curative intent and fulfilled one of two predefined inclusion criteria: (1) underwent sentinel lymph node biopsy, to evaluate the prognostic significance of ITCs; or (2) were registered with FIGO (2009) stage IIIC1–IIIC2 disease (lymph node-positive cases), to evaluate the prognostic significance of nodal tumor burden. Accordingly, the study cohort was assembled specifically to address the two predefined study objectives and did not represent all patients who underwent surgical lymph node staging during the study period.

Patients were excluded if they had nonepithelial uterine malignancies (e.g. sarcoma, neuroendocrine carcinoma), carcinoma in situ, incidental low-grade endometrial cancer in the setting of advanced ovarian cancer, had received neoadjuvant chemotherapy, or underwent palliative surgery. Patients without lymph node assessment or those treated outside the Stockholm–Gotland region were also excluded.

The study size was determined by inclusion of all eligible patients in the population-based registry during the study period.

### Exposure

The primary exposure was lymph node metastasis subtype. Metastases were classified according to the American Joint Committee on Cancer criteria [23]: macrometastases (> 2.0 mm), micrometastases (> 0.2–2.0 mm or > 200 cells), and ITCs (≤ 0.2 mm and ≤ 200 cells). Bulky metastases were defined as clinically apparent nodal disease identified intraoperatively by the surgeon and subsequently confirmed pathologically.

Sentinel lymph nodes were processed according to institutional ultrastaging protocols. When the maximum diameter of the sentinel lymph node exceeded 1 mm, the node was serially sectioned and examined in five sections at three different levels, approximately 200 μm apart, using hematoxylin and eosin staining. Immunohistochemistry (e.g. cytokeratin markers) was performed on the deepest level to detect low-volume disease.

### Control groups

In analyses of ITCs, patients without nodal dissemination served as controls. Patients with micrometastases, macrometastases, and bulky metastases were compared mutually.

### Outcomes

The primary outcomes were (1) overall survival (OS) in patients with ITCs versus no nodal dissemination and (2) OS according to lymph node metastasis subtype. OS was defined as time from diagnosis to death from any cause or censoring on July 28, 2025. Survival status was obtained through the Swedish Population Register.

Cancer-specific survival (CSS) was additionally evaluated and defined as time from diagnosis to death from endometrial cancer. Cause of death was determined by review of hospital medical records, including the physician-reported cause of death.

### Covariates

Covariates were predefined based on clinical relevance and prior literature and included age, American Society of Anesthesiologists (ASA) physical status classification, FIGO 2009 stage, histologic subtype, lymphovascular space invasion (LVSI), depth of myometrial invasion, p53 status assessed by immunohistochemistry, year of treatment, and adjuvant treatment.

### Statistical analyses

Baseline patient, tumor, and treatment characteristics were summarized according to lymph node dissemination status. Categorical variables were presented as numbers and percentages (*n*, %) and associations between categorical variables and lymph node dissemination status were assessed using Pearson’s chi-squared test or Fisher’s exact test, as appropriate. Continuous variables were presented as medians with interquartile ranges [IQRs] and means with standard deviations and were compared across lymph node dissemination groups using the Kruskal-Wallis rank-sum test. Missing data were minimal across variables and were reported descriptively without imputation.

Univariable and multivariable Cox proportional hazards regression models were used to evaluate associations between baseline characteristics and OS. In the first multivariable model evaluating patients with stage I–II disease undergoing sentinel lymph node biopsy (no dissemination vs. ITCs), OS was adjusted for age (≤ 68, > 68 years), ASA physical status (I–II, III–IV), year of surgery, histologic subtype (low-grade endometrioid, high-grade endometrioid/non-endometrioid), depth of myometrial invasion (< 50%, ≥ 50%), LVSI (absent, present), p53 mutation status (p53 wild type, p53 mutation, unknown), adjuvant treatment (none, chemotherapy and radiation therapy, chemotherapy alone and/or other treatment), and FIGO stage (I vs. II).

A second multivariable Cox proportional hazards model was fitted to evaluate the association between lymph node metastasis subtype (bulky metastases, macrometastases, and micrometastases) and OS, adjusted for age (≤ 68, > 68 years), ASA physical status (I–II, III–IV), year of surgery, histologic subtype (low-grade endometrioid, high-grade endometrioid/non-endometrioid), depth of myometrial invasion (< 50%, ≥ 50%), LVSI (absent, present), and p53 mutation status (p53 wild type, p53 mutation, unknown). Adjuvant treatment was not included in this model due to near-universal use in node-positive disease and the risk of confounding by indication.

Corresponding univariable and multivariable Cox proportional hazards regression models were fitted to evaluate CSS. For CSS analyses, patients were followed from the date of diagnosis until death from endometrial cancer. Deaths due to causes other than endometrial cancer were treated as censoring events.

Exploratory subgroup analyses were conducted by stratifying on p53 mutation status (p53 wild type or p53 mutation). Separate Cox proportional hazards regression models were fitted within each subgroup to assess whether the association between lymph node dissemination and OS differed according to molecular subtype.

The proportional hazards assumption was assessed using standard diagnostic methods. Variables violating the assumption were included as stratification factors in the Cox models.

Hazard ratios (HRs) with 95% confidence intervals (CIs) and Wald *p*-values were reported. Two-sided *p*-values < 0.05 were considered statistically significant. Median follow-up time was estimated using the reversed Kaplan-Meier method. All statistical analyses were performed using R version 4.4.1 [24].

## Results

**Figure 1:**
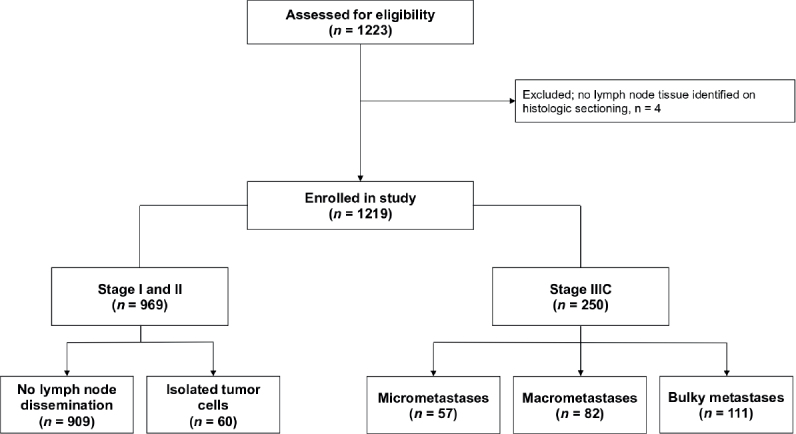
Flowchart of patient selection from the Swedish Quality Registry of Gynecologic Cancer, 2010–2024^*^. Patients were identified according to two predefined inclusion criteria: (1) patients who underwent sentinel lymph node biopsy and (2) patients registered in the Swedish Quality Registry of Gynecologic Cancer with FIGO (2009) stage IIIC1–IIIC2 disease. Accordingly, the study cohort does not represent all patients who underwent surgical lymph node staging during the study period. The FIGO (2009) stage I–II cohort comprised patients undergoing sentinel lymph node biopsy and includes patients with isolated tumor cells, which do not constitute nodal metastases for staging purposes. ^*^Coverage against the Swedish National Cancer Registry exceeded 98%.

Between January 2010 and June 2024, 1223 patients fulfilling one of the two predefined inclusion criteria were identified from the SQRGC (Figure 1). After excluding four patients without lymph node tissue in sentinel node specimens, 1219 patients were included. Overall, 25% (*n* = 310) of the study cohort had lymph node dissemination. Of these, 250 patients had metastatic disease corresponding to FIGO 2009 stage IIIC, including 36% (*n* = 111) with bulky metastases (clinically apparent during surgery), 26% (*n* = 82) with macrometastases, and 18% (*n* = 57) with micrometastases; the remaining 19% (*n* = 60) had ITCs (Figure 1). Among patients with FIGO stage IIIC, 44% had bulky metastases, 33% had macrometastases, and 23% had micrometastases. Median follow-up was 70 months (IQR, 49–109) for patients with FIGO stage IIIC disease and 50 months (IQR, 32–70) for those with stage I–II disease. Patients without lymph node dissemination were identified exclusively through the sentinel lymph node biopsy cohort, whereas all patients with FIGO stage IIIC disease were included irrespective of the method of lymph node assessment as these represented the predefined cohort for the nodal tumor burden analysis.

During follow-up, 152 patients died. Endometrial cancer was the cause of death in 121 (80%), whereas 9 (6%) died from another malignancy and 15 (10%) from other causes. The cause of death was unknown for 7 patients (5%) (Table S1).

Baseline patient, tumor, and treatment characteristics according to lymph node dissemination status are presented in Table 1. The proportion of low-grade histologic subtype and superficial myometrial invasion was higher among patients with lower extents of lymph node dissemination (*p* < 0.001). In contrast, the presence of LVSI, p53 mutation, and use of adjuvant therapy were more frequent with increasing extent of nodal disease (*p* < 0.001). The distribution of lymph node dissemination subtypes also differed by year of treatment (*p* < 0.001). This pattern reflects both the phased implementation of sentinel lymph node biopsy following its introduction into routine clinical practice in 2017 and the predefined study design, whereby patients without nodal dissemination were identified exclusively through the sentinel lymph node biopsy cohort, whereas patients from the earlier years primarily comprised those registered with FIGO (2009) stage IIIC disease. The lower number of patients in 2024 reflects that inclusion ended on June 19, 2024.

**Table 1:** Baseline patient, tumor, and treatment characteristics according to lymph node dissemination status in the predefined analytic cohort of patients with endometrial cancer, Stockholm–Gotland region, 2010–2024*.

Characteristic	Bulky metastases^a^ *n* = 111	Macro metastases^a^ *n* = 82	Micro metastases^a^ *n* = 57	Isolated tumor cells^a^ *n* = 60	No lymph node dissemination* *n* = 909	*P* ^b^
Age at surgery, years						0.292
Median (Q1, Q3)	68 (59, 74)	70 (64, 75)	68 (62, 76)	71 (62, 75)	68 (60, 75)	
Mean (SD)	66 (10)	69 (9)	69 (8)	69 (9)	67 (10)	
ASA physical status, no. (%)						0.503
I-II	78 (70)	60 (74)	44 (77)	46 (77)	705 (78)	
III-IV	33 (30)	21 (26)	13 (23)	14 (23)	203 (22)	
Missing	0	1	0	0	1	
Histologic type, no. (%)						< 0.001
Low-grade endometrioid carcinoma	26 (23)	30 (37)	29 (51)	38 (63)	663 (73)	
High-grade and non-endometrioid^c^	85 (77)	52 (63)	28 (49)	22 (37)	246 (27)	
Myometrial invasion, no. (%)						<0.001
< 50%	28 (25)	23 (28)	28 (49)	31 (52)	728 (80)	
≥ 50%	83 (75)	59 (72)	29 (51)	29 (48)	181 (20)	
LVSI, no. (%)						< 0.001
Present	78 (70)	66 (80)	34 (60)	21 (35)	109 (12)	
Absent	32 (29)	14 (17)	23 (40)	39 (65)	796 (88)	
Missing	1 (0.9)	2 (2.4)	0 (0)	0 (0)	4 (0.4)	
p53 status, no. (%)						< 0.001
p53 mutation	38 (34)	36 (44)	16 (28)	13 (22)	143 (16)	
p53 wild type	68 (61)	43 (52)	41 (72)	44 (73)	730 (80)	
Missing	5 (5)	3 (4)	0 (0)	3 (5)	36 (4)	
Year of surgery, no. (%)						< 0.001
2011	1 (1)	0 (0)	0 (0)	0 (0)	0 (0)	
2012	5 (5)	2 (2)	2 (4)	0 (0)	0 (0)	
2013	7 (6)	4 (5)	1 (2)	0 (0)	0 (0)	
2014	11 (10)	5 (6)	2 (4)	0 (0)	0 (0)	
2015	7 (6)	5 (6)	0 (0)	0 (0)	0 (0)	
2016	9 (8)	8 (10)	2 (4)	0 (0)	0 (0)	
2017	10 (9)	11 (13)	4 (7)	8 (13)	32 (4)	
2018	4 (4)	6 (7)	5 (9)	2 (3)	69 (8)	
2019	18 (16)	2 (2)	7 (12)	5 (8)	136 (15)	
2020	12 (11)	13 (16)	13 (23)	5 (8)	150 (17)	
2021	7 (6)	7 (9)	3 (5)	14 (23)	159 (17)	
2022	8 (7)	9 (11)	7 (12)	5 (8)	138 (15)	
2023	6 (5)	8 (10)	6 (11)	10 (17)	166 (18)	
2024	6 (5)	2 (2)	5 (9)	11 (18)	59 (7)	
Adjuvant treatment**, no. (%)						< 0.001
No	11 (10)	4 (5)	5 (9)	32 (53)	813 (89)	
RT	1 (1)	2 (2)	1 (2)	0 (0)	0 (0)	
CHT + RT or RCT	81 (73)	71 (87)	45 (79)	11 (18)	1 (0)	
CHT	14 (13)	4 (5)	6 (11)	17 (28)	95 (10)	
Endocrine therapy	1 (1)	0 (0)	0 (0)	0 (0)	0 (0)	
Other combination^d^	3 (3)	1 (1)	0 (0)	0 (0)	0 (0)	

* The study cohort included two groups: (1) all patients who underwent sentinel lymph node biopsy and (2) all patients registered with FIGO (2009) stage IIIC disease (lymph node metastases). The cohort therefore does not include all patients who underwent surgical lymph node staging during the study period. Patients without lymph node dissemination could enter the study only if they had undergone sentinel lymph node biopsy. Sentinel lymph node biopsy was not routinely performed until 2017. Therefore, patients who underwent other forms of lymph node staging and had no lymph node dissemination during 2011–2016 were not included in this study. This explains why the table shows no patients without lymph node dissemination during 2011–2016; it does not mean that no patients underwent lymph node staging during these years. No patients were registered with FIGO (2009) stage IIIC disease in 2010; therefore, that year is not represented in the table.

a Metastatic lymph node involvement is classified as follows: Bulky metastases (clinically apparent palpable and visible during surgery), macrometastases (> 2 mm), micrometastases (> 0.2 and ≤ 2 mm and/or > 200 cells), and isolated tumor cells (ITCs) (≤ 0.2 mm and ≤ 200 cells).

b Kruskal-Wallis rank sum test; Pearson’s Chi-squared test.

c Includes: serous, clear cell, undifferentiated, mixed, mesonephric-like, gastrointestinal mucinous type carcinoma, and carcinosarcomas.

d e.g. CHT + immunotherapy.

** Adjuvant treatment after surgery was standard of care for bulky, macro-, and micrometastatic nodal disease during the study period. Twenty-three percent of patients with isolated tumor cells received adjuvant treatment explicitly due to isolated tumor cells.

Percentages may not total 100% because of rounding. Q, quartile; SD, Standard Deviation; LN, lymph node; ASA, American Society of Anaesthesiologists physical status classification; LVSI, lymphovascular space invasion; RT, radiotherapy; RCT, concomitant radiation chemotherapy; CHT, chemotherapy.

**Figure 2:**
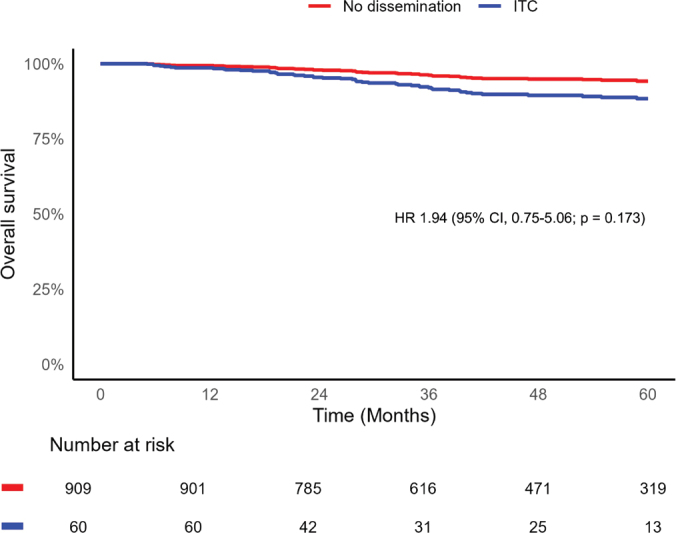
Adjusted overall survival in patients with isolated tumor cells compared with those without lymph node dissemination. Red line: No lymph node dissemination. Blue line: Isolated tumor cells in lymph node. Adjusted for: age (≤ 68 vs. > 68), year of surgery, histologic subtype (low-grade endometrioid vs. high-grade endometrioid and non-endometrioid), p53 status (p53 mutation vs. p53 wild type vs. unknown), adjuvant treatment (none vs. chemotherapy and radiation therapy vs. chemotherapy alone and/or other treatment), ASA physical status (I-II vs. III-IV), myometrial invasion (< 50% vs. ≥ 50%). The variables LVSI and FIGO stage (I and II) did not meet the proportional hazards assumption and were therefore included as strata. ITC, isolated tumor cell; CI, confidence interval; HR, hazard ratio; ASA, American Society of Anesthesiologists; LVSI, lymphovascular space invasion.

**Figure 3:**
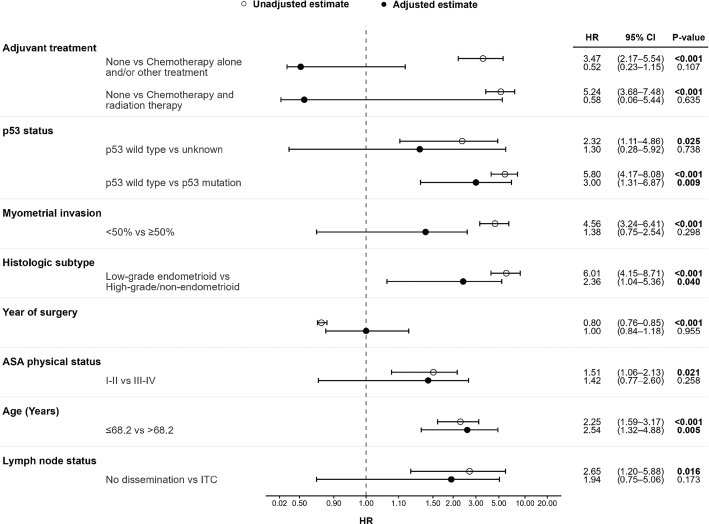
Forest plot of univariable and multivariable Cox regression analyses of the hazard of death in patients with endometrial cancer with isolated tumor cells compared with those without lymph node dissemination. White circle: Unadjusted estimate. Black circle: Adjusted estimate. Adjusted analyses included age (≤ 68 vs. > 68), year of surgery, histologic subtype (low-grade endometrioid vs. high-grade endometrioid and non-endometrioid), p53 status (p53 mutation vs. p53 wild type vs. unknown), adjuvant treatment (none vs. chemotherapy and radiation therapy vs. chemotherapy alone and/or other treatment), ASA physical status (I-II vs. III-IV) and myometrial invasion (< 50% vs. ≥ 50%). The variables LVSI and FIGO stage (I and II) did not meet the proportional hazards assumption and were therefore included as strata. ITC, isolated tumor cell; CI, confidence interval; HR, hazard ratio; ASA, American Society of Anesthesiologists; LVSI, lymphovascular space invasion.

**Figure 4:**
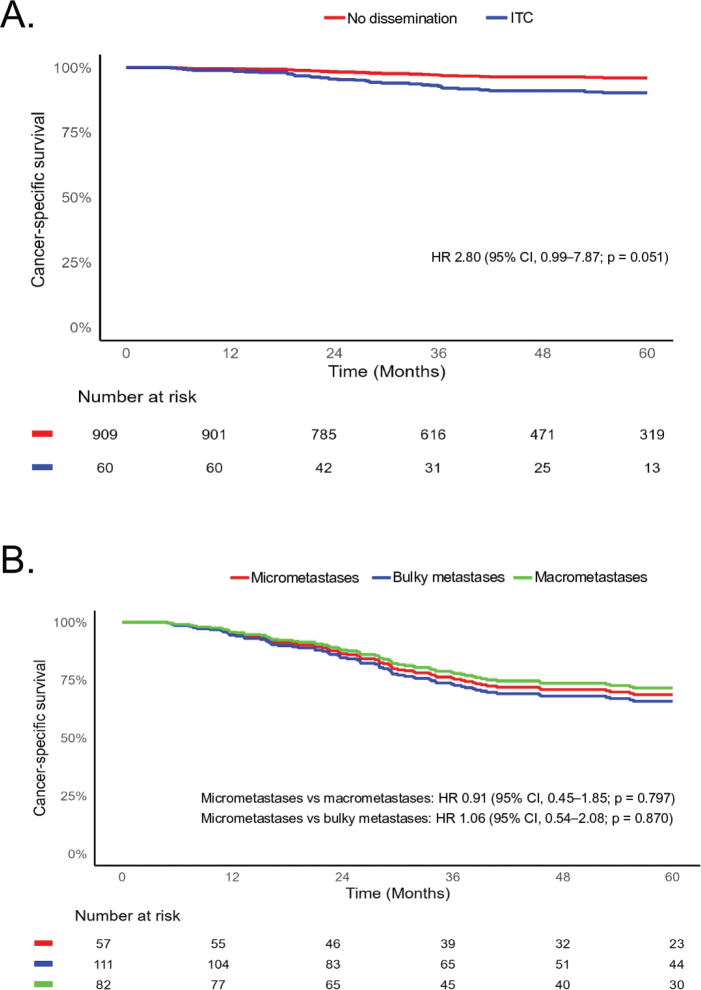
Adjusted cancer-specific survival according to lymph node dissemination. (A) Adjusted cancer-specific survival among patients with isolated tumor cells and patients without nodal dissemination. (B) Adjusted cancer-specific survival according to lymph node metastasis subtype (micrometastases, macrometastases, and bulky metastases) among patients with FIGO stage IIIC disease. Cancer-specific survival was defined as time from diagnosis to death from endometrial cancer, with deaths from other causes treated as censoring events. Survival estimates were derived from multivariable Cox proportional hazards regression models. Panel A was adjusted for age, ASA physical status, year of surgery, histologic subtype, depth of myometrial invasion, p53 status, and adjuvant treatment, with stratification by FIGO stage (2009) and LVSI. Panel B was adjusted for age, ASA physical status, year of surgery, histologic subtype, LVSI, and p53 status, with stratification by depth of myometrial invasion. Numbers at risk are shown below each panel. ITC, isolated tumor cell; CI, confidence interval; HR, hazard ratio; ASA, American Society of Anesthesiologists; LVSI, lymphovascular space invasion.

In the comparison of patients with ITCs and node-negative patients, adjusted 5-year OS was 88% (95% CI, 81–96) and 94% (95% CI, 92–96), respectively (Figure 2). No significant difference in survival was observed (HR 1.94, 95% CI, 0.75–5.06; *p* = 0.173). The point estimate suggested a possible increased risk, but the CI was wide, reflecting the limited number of events. Adjuvant treatment was more frequently administered in patients with ITCs (47% vs. 11%), and 23% received treatment specifically due to ITCs (Table 1). Crude survival estimates are shown in (Figure S1). In multivariable analysis, factors associated with worse survival included age > 68 years (HR 2.54, 95% CI, 1.32–4.88; *p* = 0.005), high-grade endometrioid or non-endometrioid histologic subtype (HR 2.36, 95% CI, 1.04–5.36; *p* = 0.040), and p53 mutation (HR 3.00, 95% CI, 1.31–6.87; *p* = 0.009) (Figure 3). No absolute differences in adjusted 5-year OS were observed (difference 6% [95% CI, −2 to 13]). Cancer-specific survival analyses yielded similar findings (Figure 4A). Adjusted 5-year CSS was 90% (95% CI, 84–97) among patients with ITCs and 96% (95% CI, 95–98) among node-negative patients. No statistically significant difference was observed after multivariable adjustment (HR 2.80, 95% CI, 0.99–7.87; *p* = 0.051).

**Figure 5:**
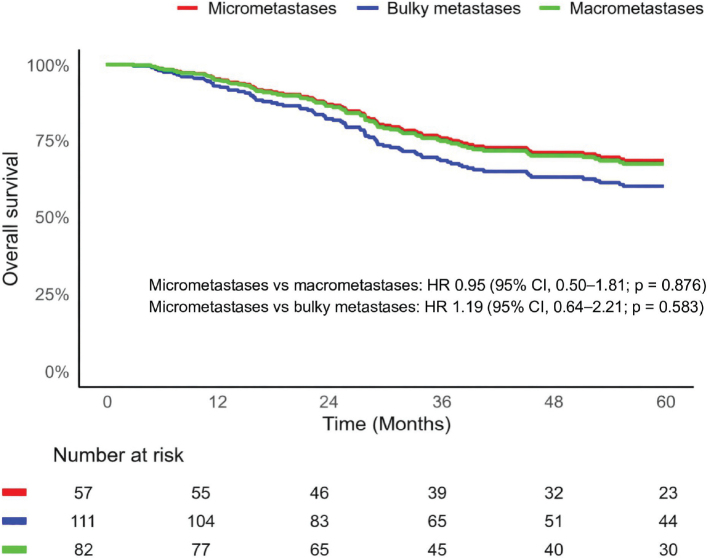
Adjusted overall survival according to lymph node metastasis subtype in patients with endometrial cancer. Red line: Micrometastases. Blue line: Bulky metastases. Green line: Macrometastases. Adjusted for: age (≤ 68 vs. > 68), year of surgery, histologic subtype (low-grade endometrioid vs. high-grade endometrioid and non-endometrioid), LVSI, p53 status (p53 mutation vs. p53 wild type vs. unknown). The variables ASA physical status and myometrial invasion did not meet the proportional hazards assumption and were therefore included as strata. CI, confidence interval; HR, hazard ratio; ASA, American Society of Anesthesiologists; LVSI, lymphovascular space invasion.

Among patients with lymph node metastases (FIGO stage IIIC), the adjusted 5-year OS was 60% (95% CI, 51–70) for bulky metastases, 67% (95% CI, 58–77) for macrometastases, and 69% (95% CI, 55–82) for micrometastases (Figure 5). No significant differences were observed, and survival was similar between micrometastases and macrometastases (HR 0.95, 95% CI, 0.50–1.81; *p* = 0.876), or between micrometastases and bulky metastases (HR 1.19, 95% CI, 0.64–2.21; *p* = 0.583). Crude survival estimates are shown in (Figure S1). In multivariable analysis, age > 68 years (HR 1.95, 95% CI, 1.25–3.06; *p* = 0.003) and p53 mutation (HR 2.85, 95% CI, 1.66–4.88; *p* < 0.001) were associated with worse survival (Table 2). Cancer-specific survival analyses yielded similar findings (Figure 4B). Adjusted 5-year CSS was 66% (95% CI, 58–74) for bulky metastases, 72% (95% CI, 63–81) for macrometastases, and 69% (95% CI, 55–82) for micrometastases. No statistically significant differences were observed between micrometastases and macrometastases (HR 0.91, 95% CI, 0.45–1.85; *p* = 0.797) or between micrometastases and bulky metastases (HR 1.06, 95% CI, 0.54–2.08; *p* = 0.870).

**Table 2:** Association between lymph node metastasis subtypes (FIGO stage IIIC) and overall survival in patients with endometrial cancer in the Stockholm–Gotland region, 2010–2024.

Characteristic	Events (%)/*n*	Unadjusted			Adjusted^a^		
		HR	95% CI	*P*	HR	95% CI	*P*
Lymph node status							
Micrometastases	15 (26)/57	1	1		1	1	
Bulky metastases	50 (45)/111	1.80	(1.01, 3.22)	0.045	1.19	(0.64, 2.21)	0.583
Macrometastases	33 (40)/82	1.62	(0.88, 2.98)	0.122	0.95	(0.50, 1.81)	0.876
Age (Years)							
≤ 68	34 (28)/123	1	1		1	1	
> 68	64 (50)/127	2.25	(1.59, 3.17)	< 0.001	1.95	(1.25, 3.06)	0.003
Year of surgery		0.80	(0.76, 0.85)	< 0.001	0.99	(0.92, 1.07)	0.826
Histologic type							
Low-grade endometrioid carcinoma	18 (21)/85	1	1		1	1	
High-grade endometrioid and non-endometrioid	80 (48)/165	6.01	(4.15, 8.71)	< 0.001	1.25	(0.64, 2.44)	0.508
LVSI							
Absent	22 (32)/69	1	1		1	1	
Present	73 (41)/178	5.10	(3.66, 7.12)	< 0.001	1.15	(0.70, 1.88)	0.576
p53 status							
p53 wild type	36 (24)/152	1	1		1	1	
p53 mutation	56 (62)/90	5.80	(4.17, 8.08)	< 0.001	2.85	(1.66, 4.88)	< 0.001
Unknown	6 (75)/8	2.32	(1.11, 4.86)	0.025	2.05	(0.67, 6.28)	0.208

a Adjusted for all included variables. The variables ASA physical status and Myometrial invasion did not meet the proportional hazards regression and were therefore included as strata. HR, hazard ratio; CI, confidence interval; LVSI, lymphovascular space invasion; ASA, American Society of Anesthesiologists.

In exploratory subgroup analyses stratified by p53 mutation status (Figures S2 and S3), no clear differences in survival were observed between patients with ITCs and node-negative patients among p53 wild type tumors, whereas survival was poorer in p53 mutated tumors. Among node-positive patients, only minor differences were observed across nodal subtypes in p53 wild type tumors, while survival was uniformly poor in p53 mutated tumors. These patterns were consistent with the main analyses, suggesting that survival differences were primarily related to tumor biology rather than nodal tumor burden.

## Discussion and conclusion

In this large population-based cohort of patients with endometrial cancer, no clear difference in OS was observed between patients with ITCs and those without nodal dissemination. Nevertheless, nearly half of patients with ITCs received adjuvant treatment, and 23% were treated specifically because of their presence. In a separate analysis of node-positive patients with FIGO stage IIIC disease, no clear differences in OS were observed according to lymph node metastasis subtype. Nearly half of FIGO stage IIIC cases had clinically apparent bulky nodal metastases detectable at surgery. Across both analyses, older age and p53 mutation status were independently associated with survival, suggesting that both clinical and molecular factors may be important determinants of prognosis relative to the extent of nodal disease.

The role of lymph node assessment in endometrial cancer remains debated, particularly with respect to its impact on patient outcomes. While sentinel lymph node biopsy has been widely adopted to reduce surgical morbidity, its primary justification is diagnostic rather than therapeutic. Whether expanded lymph node assessment and ultrastaging are associated with improved patient outcomes remains uncertain [3]. Ultrastaging has increased the detection of very low-volume nodal disease, particularly ITCs [14]. Similar developments have been observed in other malignancies, where increasingly sensitive diagnostic techniques were introduced ahead of prospective evidence of clinical benefit, leading to clinical uncertainty and heterogeneous management [25–27].

In this study, the comparison between patients with ITCs and those without nodal dissemination was based on the predefined sentinel lymph node biopsy cohort, comprising nearly 1000 patients, whereas the analysis of nodal tumor burden included all patients registered with FIGO (2009) stage IIIC disease during the study period. This reflects the implementation of sentinel lymph node mapping with ultrastaging in routine clinical practice at our institution from 2017 onward. The increasing detection of ITCs over time reflects changes in staging and pathological assessment rather than a biological shift in disease presentation.

The prevalence of ITCs was low, consistent with prior reports [28]. In crude analyses, patients with ITCs appeared to have poorer survival; however, this association was attenuated after multivariable adjustment, and no significant association was observed. Confidence intervals were wide, and clinically relevant differences cannot be excluded. These findings are consistent with previous studies, which have generally not demonstrated statistically significant differences in OS between patients with ITCs and those with node-negative disease, although results remain heterogeneous and are often limited by confounding, including differential use of adjuvant therapy [20, 21]. Taken together, our findings do not provide evidence for an independent association between ITCs and OS.

Clinical management of patients with ITCs remains heterogeneous. In our cohort, nearly half of patients with ITCs received adjuvant therapy, and 23% were treated specifically because of the presence of ITCs rather than other clinicopathologic risk factors, providing an estimate of treatment escalation attributable to ITCs in routine clinical practice. Nevertheless, most treated patients received adjuvant therapy based on uterine or other high-risk features, raising uncertainty about the incremental clinical value of detecting ITCs in guiding treatment decisions. In the absence of a clear survival benefit, these findings raise questions about the justification for treatment escalation based solely on ITCs, particularly given the well-recognized acute and long-term toxicities of adjuvant therapy [15–19, 29–32].

While lymph node metastases are generally associated with worse survival, the clinical presentation of nodal metastases within FIGO stage IIIC endometrial cancer has been poorly characterized. Nodal metastases were clinically apparent at surgery in 44% of patients, providing a population-level estimate of clinically detectable nodal disease. Despite differences in nodal disease volume and mode of detection, no statistically significant associations with OS were observed after multivariable adjustment. These findings suggest that any independent prognostic effect of nodal tumor burden may be limited after accounting for clinicopathologic and molecular factors. However, a prognostic effect of nodal tumor burden cannot be excluded, and the study may have been underpowered to detect modest differences. Previous studies have reported worse survival among patients with macro-metastases; however, available data are limited and often based on smaller cohorts or incomplete adjustment for key prognostic factors. Although larger studies have accounted for selected clinicopathologic variables, comprehensive adjustment including molecular classification has been lacking [21, 33]. Notably, no prior study has evaluated clinically apparent (bulky) nodal metastases as a separate category. Given that adjuvant therapy is standard of care for node-positive disease and was administered to nearly all patients, it was not included in the adjusted models due to limited variability and the risk of confounding by indication. Consistent with emerging evidence highlighting the prognostic importance of molecular subtype, our findings suggest that previously reported differences may largely reflect underlying tumor biology rather than nodal tumor burden per se [34].

Across both primary endpoint analyses, older age and p53 mutation status were independently associated with survival, consistent with prior reports [35–40]. In the comparison between ITCs and no nodal dissemination, aggressive histologic subtype was also associated with worse survival. While earlier FIGO staging systems were based on anatomic disease dissemination, the most recent revision incorporates molecular classification but still excludes readily defined clinical prognostic factors such as age [41]. In exploratory subgroup analyses stratified by p53 mutation status, survival appeared to be driven primarily by tumor biology rather than nodal disease extent. Although these analyses were based on small numbers, the consistency of findings across subgroups and the main analyses supports this interpretation. The subgroup analyses were not powered to detect interactions. Together, these findings highlight the importance of clinical and molecular factors for risk stratification.

Several limitations of our study merit consideration. Although this represents one of the largest population-based cohorts examining lymph node metastasis subtypes in endometrial cancer including all patients with FIGO (2009) stage IIIC disease during the entire 14-year study period to maximize the available population with nodal metastases, the number of patients with nodal disease was limited, reflecting the underlying epidemiology. This reduced the precision of survival estimates and the ability to detect modest differences. The small number of events among patients with ITCs may also have limited statistical power to detect modest differences, increasing the risk of type II error. The observed HRs – particularly for ITCs – suggest that a clinically relevant difference cannot be excluded, and the findings should therefore be interpreted with caution. In addition, residual confounding related to treatment selection and unmeasured factors cannot be excluded.

Comprehensive molecular classification was not available throughout the study period due to temporal changes in diagnostic practice; however, p53 mutation status, a strong prognostic marker, was included in the analyses. The population-based design, centralized gynecologic cancer care within a single tertiary referral center, capture of more than 98% of eligible patients, and systematic validation of registry data against medical records strengthen the robustness and internal validity of the findings. Although generalizability to healthcare systems with different referral patterns may be limited, the uniform organization of care and comprehensive follow-up within the Nordic publicly funded healthcare system provide internally valid estimates reflecting contemporary practice.

In conclusion, no clear difference in OS was observed between patients with ITCs and those without nodal dissemination in this large Swedish cohort, although modest associations cannot be excluded. Similarly, no clear differences in OS were observed across lymph node metastasis subtypes. Taken together, these findings suggest that clinical and molecular characteristics may be important determinants of prognosis relative to the extent of nodal involvement. These findings also highlight the uncertain clinical benefit of expanded nodal assessment and treatment escalation based solely on nodal disease extent. Prospective studies are needed to better define the clinical implications of very low-volume nodal disease and to determine whether management strategies based on its detection improve patient outcomes.

## Supplementary Material



## Data Availability

Individual-level data cannot be shared due to restrictions imposed by the ethical review board. Aggregated data are available from the corresponding author upon reasonable request.
