# Supplementary material for: Prognostic significance of isolated tumor cells and nodal tumor burden in endometrial cancer: a population-based cohort study
Source: Acta Oncol. 2026 Aug 11;65:45813. doi: 10.2340/1651-226X.2026.45813 (PMC13479639; doi:10.2340/1651-226X.2026.45813)
Supplement: Supplementary file 1 [file AO-65-45813-s1.pdf]

Supplementary material has been published as submitted. It has not been copyedited, or typeset by Acta Oncologica

**Figure S1. Crude overall survival for isolated tumor cells versus no lymph node dissemination (A) and by lymph node metastasis subtype (B).**

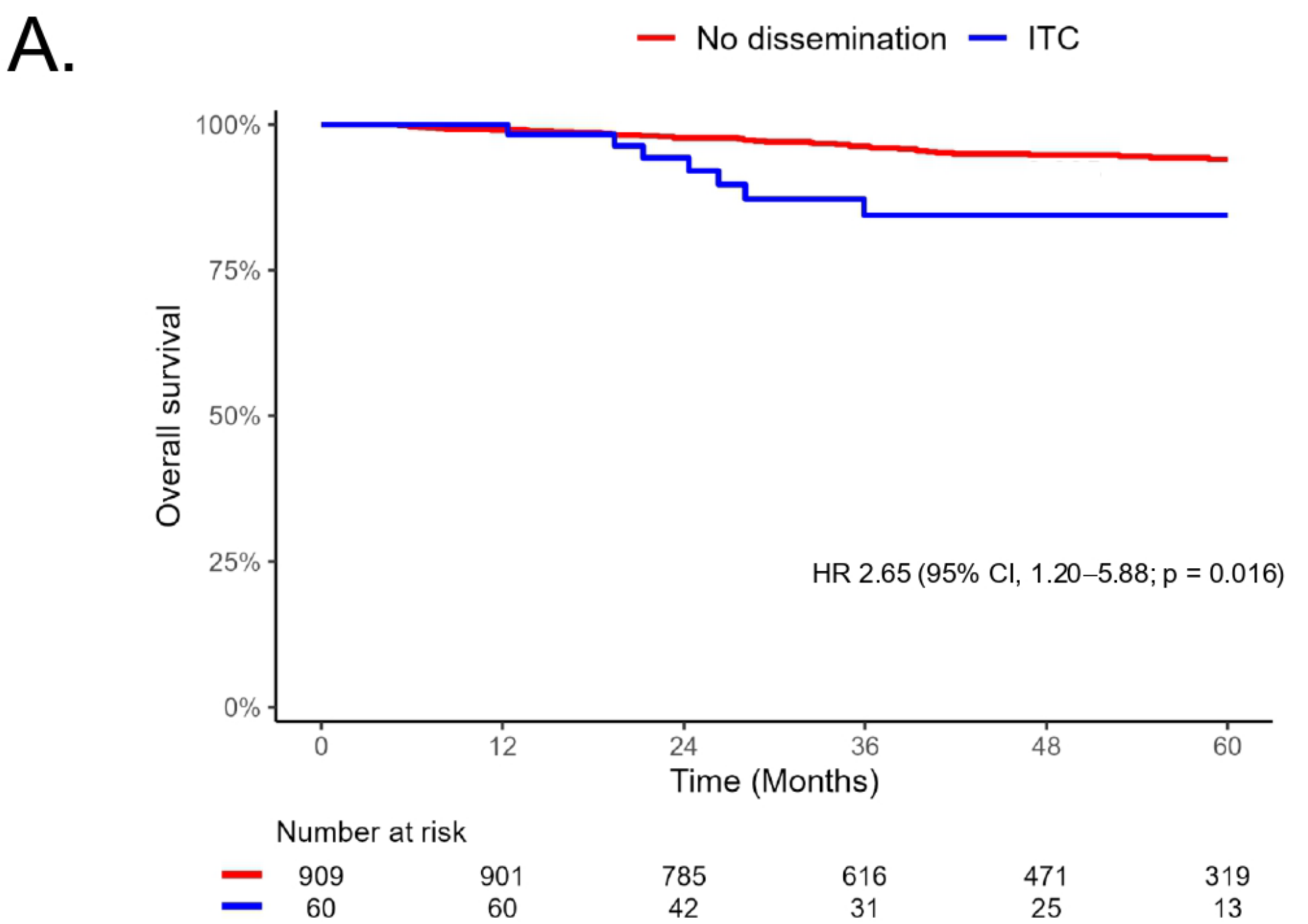


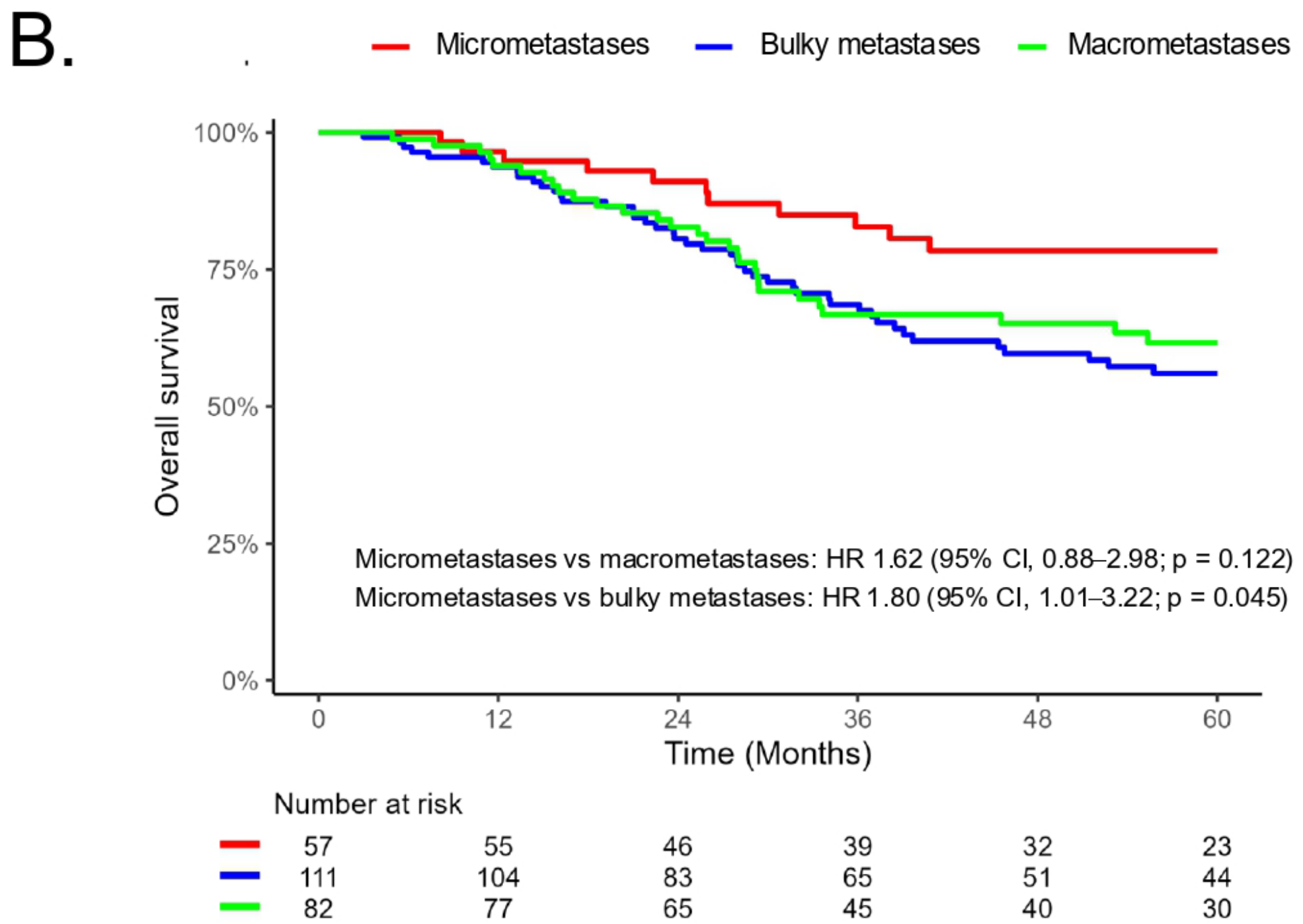

**Figure S2: p53-stratified analyses of overall survival in patients with isolated tumor cells versus no nodal dissemination**

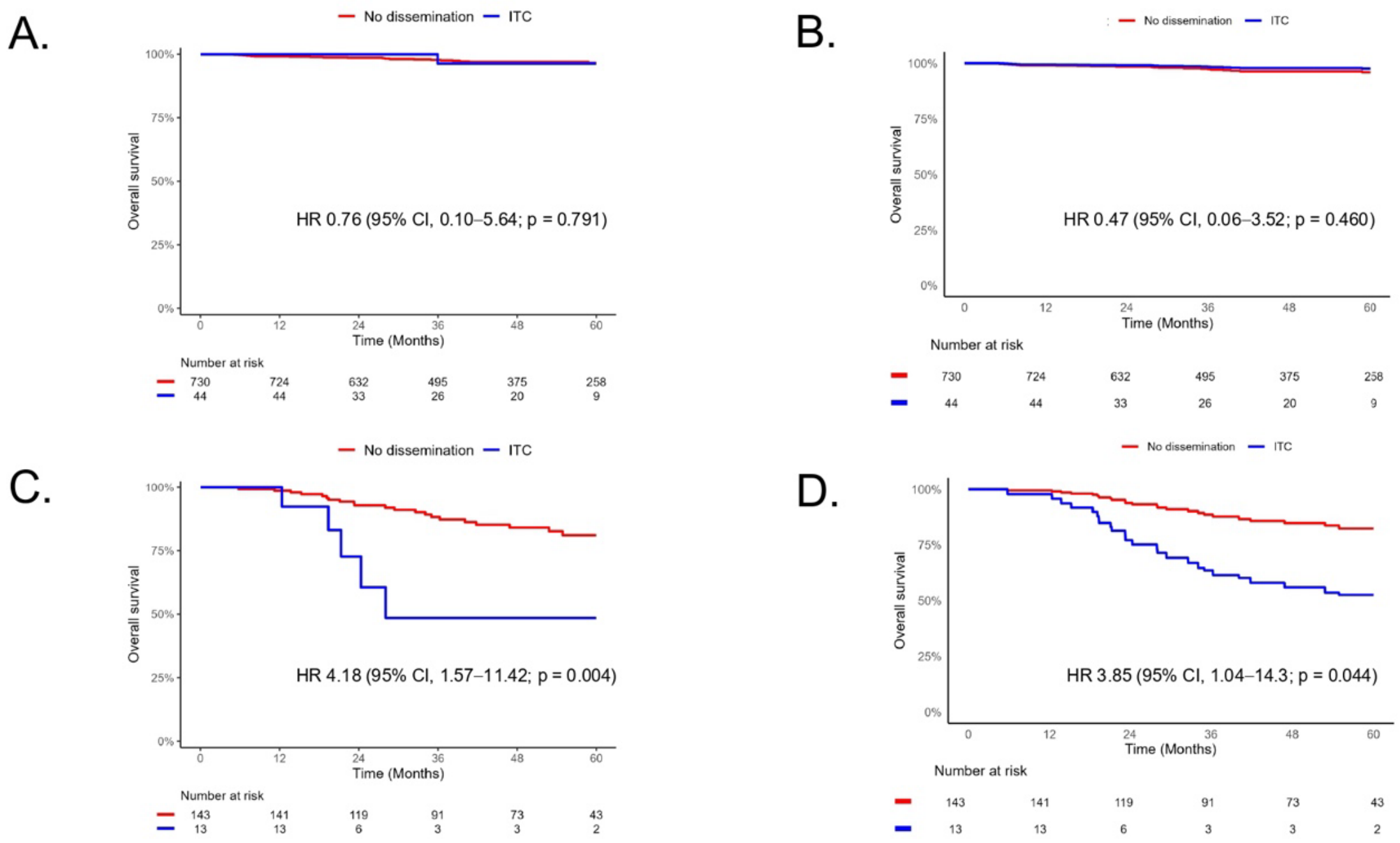


*A–B: Patients with p53 wild type tumors*

*C–D: Patients with p53 mutated tumors*

*A, C: Crude overall survival*

*B, D: Adjusted overall survival*

*In patients with p53 wild type tumors, 5-year OS was 96% (95% CI, 89–100) for ITCs and 97% (95% CI, 95–98) for node-negative patients (crude), and 98% (95% CI, 93–100) versus 96% (95% CI, 94–98), respectively (adjusted).*

*In patients with p53 mutated tumors, 5-year OS was 81% (95% CI, 74–89) for ITCs and 48% (95% CI, 25–95) for node-negative patients (crude), and 53% (95% CI, 22–83) versus 82% (95% CI, 75–90), respectively (adjusted).*

*For p53 wild type tumors, adjusted models included age, year of surgery, LVSI, ASA physical status, histologic subtype, myometrial invasion, and FIGO stage; adjuvant treatment did not meet the proportional hazards assumption and was included as a stratification variable.*

*For p53 mutated tumors, adjusted models included age, ASA physical status, year of surgery, LVSI, myometrial invasion, and adjuvant treatment; histologic subtype and FIGO stage did not meet the proportional hazards assumption and were included as stratification variables.*

**Figure S3:** p53-stratified analyses of overall survival according to nodal tumor burden in patients with endometrial cancer

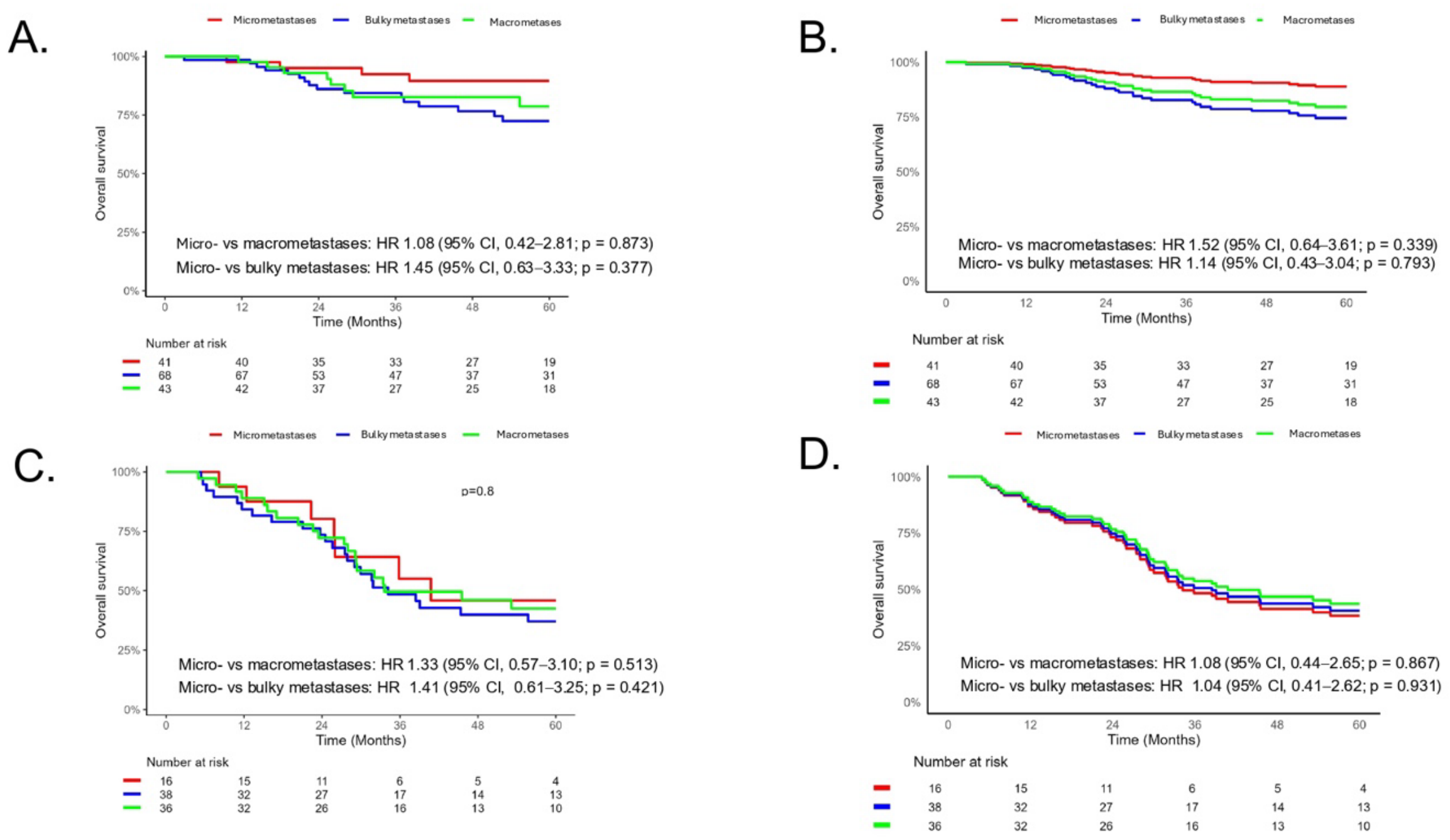


*A–B: Patients with p53 wild type tumors*

*C–D: Patients with p53 mutated tumors*

*A, C: Crude overall survival*

*B, D: Adjusted overall survival*

*In patients with p53 wild type tumors, 5-year OS was 90% (95% CI, 80–100) for micrometastases, 79% (95% CI, 66–93) for macrometastases, and 72% (95% CI, 62–85) for bulky metastases (crude), and 89% (95% CI, 79–99), 80% (95% CI, 68–92), and 75% (95% CI, 63–86), respectively (adjusted).*

*In patients with p53 mutated tumors, 5-year OS was 46% (95% CI, 25–85) for micrometastases, 43% (95% CI, 29–63) for macrometastases, and 37% (95% CI, 24–57) for bulky metastases (crude), and 38% (95% CI, 13–63), 44% (95% CI, 28–59), and 41% (95% CI, 27–55), respectively (adjusted).*

*For p53 wild type tumors, adjusted models included age, year of surgery, LVSI, ASA physical status, and histologic subtype; myometrial invasion did not meet the proportional hazards assumption and was included as a stratification variable.*

*For p53 mutated tumors, adjusted models included age, year of surgery, and LVSI; ASA physical status, myometrial invasion, and histologic subtype did not meet the proportional hazards assumption and were included as stratification variables*

**Supporting Information Table S1. Causes of death among deceased patients during follow-up.**

| Cause of death | No. (%) of deaths |
|---|---|
| Endometrial cancer | 121 (80) |
| Other cancer | 9 (6) |
| Other causes | 15 (10) |
| Cardiovascular disease | 5 (3) |
| Multiorgan failure following medical or surgical complications | 3 (2) |
| Neurological disease | 3 (2) |
| Gastrointestinal/hepatobiliary disease | 2 (1) |
| Uncertain medical event (cardiac arrest during immunotherapy; relationship uncertain) | 1 (1) |
| Uncertain cause of death (possible suicide) | 1 (1) |
| *Unknown*[*] | 7 (5) |

*Follow-up censored on July 28, 2025.*

[*]Cause of death could not be determined because the death certificate was unavailable through the regional electronic medical record system. This included patients who had moved to another healthcare region or whose death certificate had been completed by healthcare providers not connected to the regional electronic medical record system.

Percentages may not sum to 100 because of rounding.
